# Mucinous Bladder Adenocarcinoma: Case Report and Literature Review

**DOI:** 10.1155/2015/783109

**Published:** 2015-09-01

**Authors:** Bruno Mello R. Santos, Julia Duarte de Souza, Rachel Silviano Brandão Correa Lima, Enaldo Melo de Lima

**Affiliations:** ^1^UFMG School of Medicine, 31270-901 Belo Horizonte, MG, Brazil; ^2^Hospital Mater Dei, 30140-093 Belo Horizonte, MG, Brazil; ^3^Pelvic Floor Dysfunction Unit, Mater Dei Hospital, 30140-093 Belo Horizonte, MG, Brazil; ^4^Oncology Unit, Mater Dei Hospital, 30140-093 Belo Horizonte, MG, Brazil

## Abstract

Primary mucinous adenocarcinoma is an extremely rare type of bladder cancer, with aggressive behavior and poor response to chemotherapy and radiotherapy. The symptoms are similar to those of other bladder tumors. Surgery is the main treatment and remains the only curative option. There may be a progression from mucinous metaplasia to mucinous adenoma and then mucinous adenocarcinoma. We present the case of a 40-year-old woman with recurrent lower urinary tract infections, submitted to imaging tests, which showed a bladder tumor. After transurethral resection, pathology showed intestinal mucinous carcinoma. Metastatic work-up was negative. New surgical procedure showed metaplasia but no recurrence of the carcinoma. The patient is now using antibiotic prophylaxis and will undergo a cystoscopy every 3 months and computed tomography in one year.

## 1. Background

Adenocarcinoma represents 0.5 to 2% of all malignant tumors of the bladder, and the mucinous subtype is extremely rare as a primary vesical lesion [1]. It is more common in patients over 50 years old. It is believed that there is a progression pattern from mucinous metaplasia to mucinous adenoma and then mucinous adenocarcinoma; however, no study has confirmed this hypothesis [2]. Adult vesical exstrophy and persistent urachal remnants also figure among the risk factors associated with vesical adenocarcinoma. As for clinical presentation, the most common symptoms are hematuria, suprapubic pain, and voiding difficulties, similar to other bladder tumors [3, 4].

This cancer has aggressive behavior, with intramural growth, which leads to late onset of symptoms and delay in diagnosis with worse prognosis. The diagnosis is based on cystoscopy, urinary cytology, and transurethral resection of the bladder. The tumor shows a poor response to chemotherapy and radiotherapy. Surgical resection is the main therapeutic option [5, 6].

## 2. Case Report

**Figure 1:**
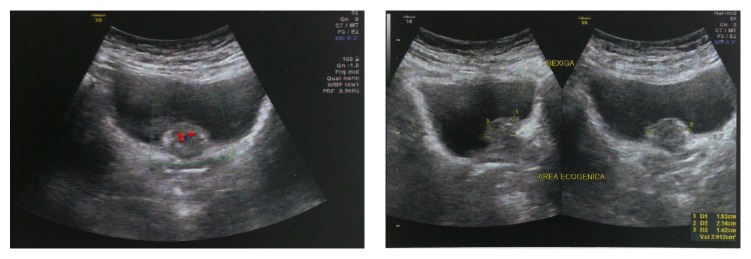
Ultrasound showing a 21 mm vegetating lesion on the bladder neck.

**Figure 2:**
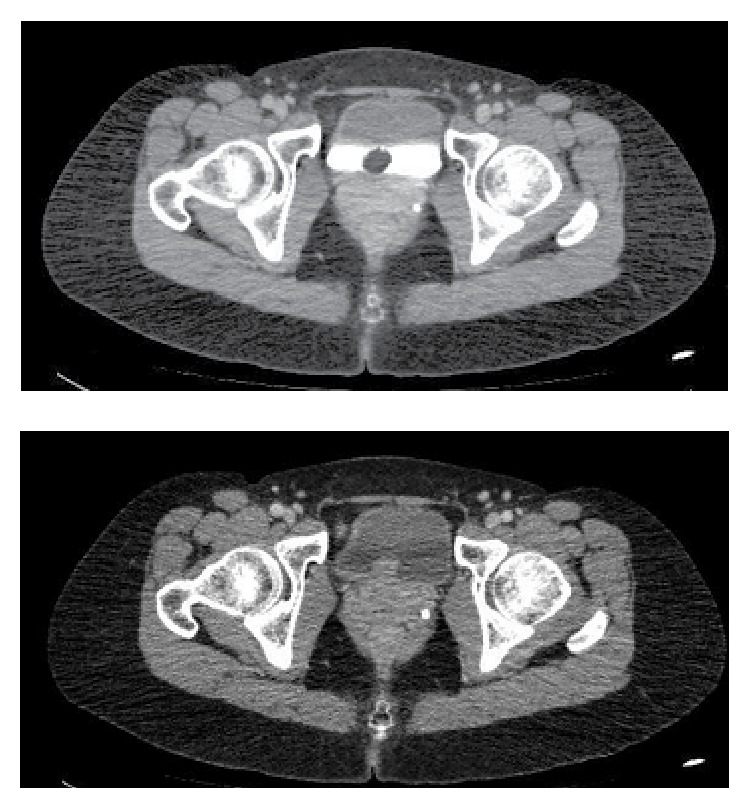
Computed tomography with intravenous contrast, showing a contrast captating solid vesical lesion.

**Figure 3:**
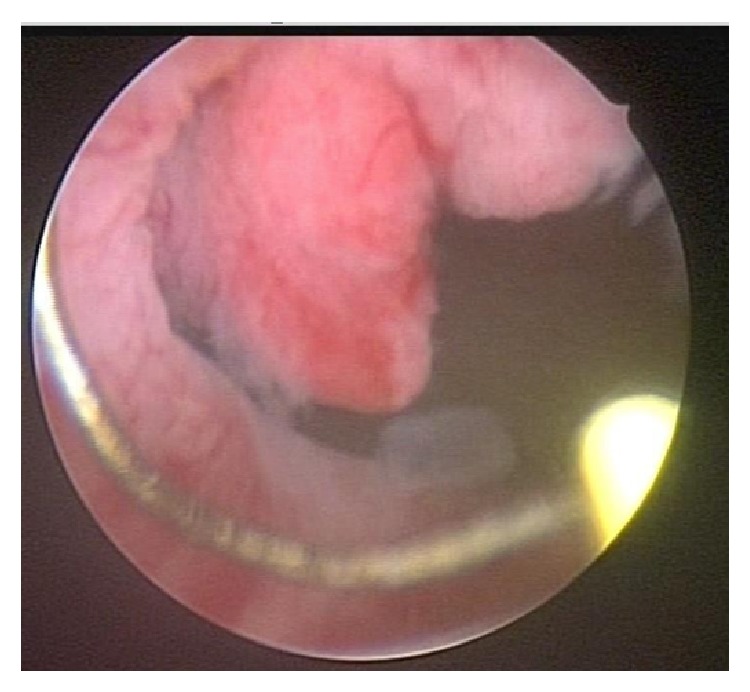
Preoperative image of the vesical lesion originating in the anterior aspect of the bladder neck, visible resection loop.

The patient is 40-year-old female, healthy, nonsmoker, with recurrent lower urinary tract infections over the last year. In the beginning of this period, and ultrasound showed no abnormalities. The next ultrasound, performed by the same radiologist one year later, showed a 21 mm vegetating bladder lesion, close to the urethra (Figure 1). The computed tomography showed the same finding: 19 mm solid bladder lesion, with contrast enhancement (Figure 2). The patient was then referred to the urologist, who performed a transurethral resection of the lesion. After routine preoperative preparation, she was submitted under spinal anesthesia, to the transurethral resection of the lesion, whose base was located in the anterior aspect of the bladder neck, sessile (Figure 3). The tumor fragments were sent to anatomical pathology and the lesion appeared to have been fully resected. After an uneventful postoperative evolution, the urethral catheter was removed and patient discharged on the second postoperative day.

**Figure 4:**
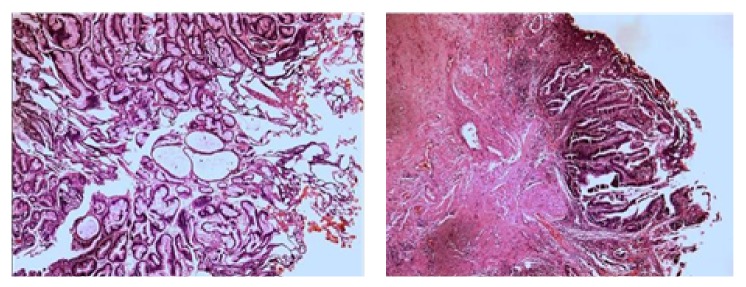
Photomicrographs of the vesical lesion, intestinal mucinous carcinoma, originated from tubular polyp with low-grade dysplasia.

Anatomical pathology showed intestinal mucinous carcinoma, originated from tubular polyp with low-grade dysplasia and suggested investigating gastrointestinal source (Figure 4). The findings were confirmed in immunohistochemistry study (positive for Anti-beta Catenin, Anti-CDX2, Anti-Cytokeratin 20, Anti-Cytokeratin 07, Anti-Cytokeratins cocktail, and Anti-p53 oncoprotein).

Due to the possibility of a primary lesion in the gastrointestinal tract, and upper digestive endoscopy, a colonoscopy and PET CT were performed, as were tumor markers (CA 19-9, CA 125, alpha-fetoprotein, and CEA), all of which without any abnormal results.

It was thus concluded that the bladder lesion was primary, which made a second transurethral resection necessary to confirm that there was no residual lesion. The resection was performed 70 days after the first one, without macroscopic evidence of recurrence. It was not performed earlier due to time taken to undergo PET CT, endoscopy, colonoscopy, and pathology review. Fragments were removed from the scar of the previous procedure. Histopathology showed fragments of mucosa and muscular layers of the vesical wall, with spots of metaplasia and spots of cystic glandular cystitis, negative for neoplastic infiltration. The patient recovered well after the second procedure. Antibiotic prophylaxis was done with nitrofurantoin (100 mg at night), to avoid any infectious process which might work as an irritating agent and possible metaplasia inductor. It was recommended that patient undergoes a cystoscopy every 3 months, and computed tomography in one year.

## 3. Discussion

When facing a mucinous bladder adenocarcinoma, it is necessary to investigate another source of cancer, since this type of tumor is extremely rare as a primary lesion. In the reported case, extensive work-up ruled out both the possibility of other sites of primary tumor and the possibility of metastasis, confirming the diagnosis of a primary bladder lesion.

Chronic irritation of the bladder mucosa often induces metaplasia (more frequently, of the squamous type). In the case of this patient, it is possible that the recurrent lower urinary tract infections worked as an irritating agent, contributing to the metaplastic transformation [7]. Another possibility is that the tumoral lesion itself favored the infections.

The classification of the intestinal metaplasia as a premalignant lesion of the adenocarcinoma is still controversial. A study by Morton et al. [8] shows significant telomere shortening in bladder intestinal metaplasia, besides cytogenetic abnormalities associated with urothelial carcinoma, endorsing the hypothesis that the metaplastic transformation precedes vesical adenocarcinoma. Other retrospective studies showed opposite results, determining that the mucinous metaplasia is not a risk factor for bladder adenocarcinoma.

The prognosis of mucinous bladder adenocarcinoma depends mainly on its stage when diagnosed and treated. When the tumor is confined to the bladder, the survival rate is 75–100%; unfortunately, less than 30% of patients are diagnosed at an early stage. In the reported case, despite the absence of symptoms, the ultrasound performed for the investigation of urinary tract infection made the early diagnosis possible. The absence of metastasis and muscular layer infiltration suggests a good prognosis.
